# Immunogenicity of a Rotavirus VP8* Multivalent Subunit Vaccine in Mice

**DOI:** 10.3390/v16071135

**Published:** 2024-07-16

**Authors:** Roberto Cárcamo-Calvo, Irene Boscá-Sánchez, Sergi López-Navarro, Noemi Navarro-Lleó, Nazaret Peña-Gil, Cristina Santiso-Bellón, Javier Buesa, Roberto Gozalbo-Rovira, Jesús Rodríguez-Díaz

**Affiliations:** 1 Department of Microbiology, School of Medicine, University of Valencia, Av. Blasco Ibáñez 15, 46010 Valencia, Spain; roberto.carcamo@uv.es (R.C.-C.); ibosca@iata.csic.es (I.B.-S.); sergi.lopez@uv.es (S.L.-N.); noemi.navarro@uv.es (N.N.-L.); nazaret.pena@uv.es (N.P.-G.); cristina.santiso@uv.es (C.S.-B.); javier.buesa@uv.es (J.B.); roberto.gozalbo@uv.es (R.G.-R.); 2 Instituto de Investigación INCLIVA, Hospital Clínico Universitario de Valencia, 46010 Valencia, Spain

**Keywords:** rotavirus, vaccines, genotypes, lineages

## Abstract

Rotavirus remains a significant public health threat, especially in low-income countries, where it is the leading cause of severe acute childhood gastroenteritis, contributing to over 128,500 deaths annually. Although the introduction of the Rotarix and RotaTeq vaccines in 2006 marked a milestone in reducing mortality rates, approximately 83,158 preventable deaths persisted, showing ongoing challenges in vaccine accessibility and effectiveness. To address these issues, a novel subcutaneous vaccine formulation targeting multiple rotavirus genotypes has been developed. This vaccine consists of nine VP8* proteins from nine distinct rotavirus genotypes and sub-genotypes (P[4], P[6], P[8]_LI_, P[8]_LIII_, P[8]_LIV_, P[9], P[11], P[14], and P[25]) expressed in *E. coli*. Two groups of mice were immunized either with a single immunogen, the VP8* from the rotavirus Wa strain (P[8]_LI_), or with the nonavalent formulation. Preliminary results from mouse immunization studies showed promising outcomes, eliciting antibody responses against six of the nine immunogens. Notably, significantly higher antibody titers against VP8* P[8]_LI_ were observed in the group immunized with the nonavalent vaccine compared to mice specifically immunized against this genotype alone. Overall, the development of parenteral vaccines targeting multiple rotavirus genotypes represents a promising strategy in mitigating the global burden of rotavirus-related morbidity and mortality, offering new avenues for disease prevention and control.

## 1. Introduction

Rotavirus (RV) remains a significant public health threat, especially in low-income countries, where it is the leading cause of severe acute childhood gastroenteritis, contributing to over 128,500 deaths annually [1]. Rotavirus is a genus belonging to the *Sedoreoviridae* family. Viral particles are spherical, with a diameter ranging from 70 to 100 nm, possessing an icosahedral capsid and lacking an envelope [2]. The classical classification system is based on the composition of its genome and the immunological reactivity of three structural proteins—VP6, VP7, and VP4 [3]. To date, nine RV species (RVA to RVD and RVF to RVJ have been officially adopted by the International Committee on Taxonomy of Viruses (ICTV)), although a putative new species has recently been proposed (RVL) [4]. The VP4 protein, which defines P genotypes, is a key determinant of rotavirus infectivity and a target for immune responses. The other outer capsid protein, VP7, is a glycoprotein (thus defining the G types). To date, a total of 42 G and 58 P types have been described within RVA species [5]. Despite the existence of multiple genotypes, the P[8] genotype is estimated to be responsible for more than 80% of infections in humans [6]. Within this genotype, phylogenetically, the following four distinct lineages have been described: P[8]_LI_, P[8]_LII_, P[8]_LIII_, and P[8]_LIV_ [7,8]. Novel genotypic combinations, such as a human G6P[6] rotavirus, have been discovered, emphasizing the importance of ongoing surveillance and characterization for vaccine design and the understanding of rotavirus diversity [9]. 

Although the introduction of the Rotarix and RotaTeq vaccines in 2006 marked a milestone in reducing mortality rates, approximately 83,158 preventable deaths persisted, showing ongoing challenges in vaccine accessibility and effectiveness [10]. These vaccines aim to induce immunity against the most common serotypes of the virus targeting the globally common rotavirus serotypes G1–G4 together with P[8], which represent a majority of the circulating strains [10]. However, more strain diversity has been identified, suggesting the need for good heterotypic protection [11,12]. In this regard, strains such as G12P[8] have emerged and become predominant, with evidence of multiple introductions and significant antigenic mismatch with vaccines, highlighting the need for ongoing surveillance and vaccine effectiveness monitoring [13]. The introduction of rotavirus vaccines has been associated with the increased diversity in rotavirus genotypes, with fluctuations in genotype dominance observed in different regions [14,15,16]. In Australia, the distribution of rotavirus genotypes varied based on the vaccine used, with G12P[8] becoming dominant in states using RotaTeq (P[8]_LII_), and equine-like G3P[8] and G2P[4] in states using Rotarix (P[8]_LI_) [14]. In the United States, the emergence of G12P[8] strains, which present antigenic epitopes differently from vaccine strains, has been noted, suggesting multiple introductions and antigenic mismatch with vaccines [13]. In the same way, Brazil experienced a rapid shift in rotavirus genotype distribution post the vaccine introduction, with G12P[8] re-emerging as the predominant genotype, raising the possibility of a G12 outbreak [17]. Japan observed a diversity in circulating rotavirus genotypes before and after vaccine introduction, with unusual strains like G2P[4], G9P[8], and G8P[8] emerging in the post-vaccination period [15]. In Iran, the most common genotype was G3P[8], with several amino acid differences in antigenic epitopes between circulating and vaccine strains, suggesting potential for the emergence of antibody-escaping mutants [18]. In India, differences in the antigenic epitopes between the circulating G1P[8] strains and vaccine strains were identified, emphasizing the need for continuous monitoring of sub-genotypic lineages [19]. Malawi did not show evidence of genotype selection due to vaccine pressure, with strain diversity likely driven by natural mechanisms [20]. In the Triângulo Mineiro region of Brazil, a single epidemic genotype G2P[4] emerged following the vaccine introduction, replacing the previous diversity of genotypes [16]. The widespread use of rotavirus vaccines may have introduced selective pressures on human rotaviruses, potentially triggering genetic and antigenic changes that could then affect vaccine effectiveness [21].

On top of the genotype diversity, vaccine failure might also be associated to differences in host microbiota [22,23,24] and host genetics factors mediated by the FUT2 (secretor) and FUT3 (Lewis) genes [25,26]. Human RVs attach to histo-blood group antigens (HBGAs) in a genotype-dependent manner [27]. The FUT2 and FUT3 polymorphisms might explain the low vaccine take [28]. This fact might have an impact in low-income countries in Africa since there is a different proportion of secretor negative and Lewis negative individuals [26]. These two factors might impair the RV replication thus decreasing the immunogenicity of the vaccines and impairing the effectiveness in those individuals taking the vaccine.

To overcome the low effectivity of replicative vaccines in low-income countries, several parenteral vaccines based on the VP8* protein of rotavirus are in development [10], using both proteins and mRNA as immunogens [29,30]. To the best of our knowledge, the new VP8* subunit vaccine formulations include a maximum of three VP8* immunogens and, due to the high diversity of circulating rotavirus, this might be insufficient to create a broad immunization able to protect against most of the circulating strains. For this reason, the aim of the present work was to assay the immunogenicity of a multivalent vaccine, including nine VP8* proteins from different genotypes and sub-genotypes (P[4], P[6], P[8]_LI_, P[8]_LIII_, P[8]_LIV_, P[9], P[11], P[14], and P[25]), in mice and compare the performance of this new formulation with a vaccine including only the VP8* protein from the cell-culture-adapted Wa strain (P[8]_LI_). Preliminary results from the mouse immunization studies showed promising outcomes, eliciting antibody responses against six of the nine immunogens. Notably, significantly higher antibody titers against VP8* P[8]_LI_ were observed in the group immunized with the nonavalent vaccine compared to the mice specifically immunized against this genotype alone.

## 2. Materials and Methods

### 2.1. Expression and Purification of Rotavirus VP8* Proteins

Nine different RVA VP8* proteins from distinct genotypes and sub-genotypes P[4], P[6], P[8]_LI_, P[8]_LIII_, P[8]_LIV_, P[9], P[11], P[14], and P[25], were produced in *E. coli* and purified by affinity chromatography as previously described by us [6,31].

### 2.2. Immunizations

Female C57BL/6J mice from Charles River Laboratories (Saint Germain Nuelles, France), aged 4–6 weeks and weighing approximately 22–24 g, were employed for the study. The mice were housed in a level 1 biosecurity animal facility. The study received approval from the Animal Experimentation Committee of the University of Valencia and the Directorate General of Agriculture, Livestock, and Fisheries (2018/VSC/PEA/0181). Three groups of C57BL/6J mice, each consisting of three individuals, were established as follows: Group 1 (E1G1), or the control group, where the mice were given only the adjuvant with PBS; Group 2 (E1G2), or the monovalent vaccine group, where the mice were immunized with the VP8* protein from the Wa strain of RV (P[8]_LI_); and Group 3 (E1G3), or the multivalent vaccine group, where they received an immunization with a combination of 9 VP8* proteins representing the various genotypes and sub-genotypes, P[4], P[6], P[8]_LI_, P[8]_LIII_, P[8]_LIV_, P[9], P[11], P[14], and P[25]. Proteins were administered at a concentration of 5 µg/mouse of each protein (this implied a final concentration of 45 µg/mouse in Group E1G3). Aluminum hydroxide hydrogel was used as an adjuvant. The protein solution in PBS 1X was prepared in a 1:1 ratio with the adjuvant and injected subcutaneously in a 100 µL inoculum. Immunizations were repeated three times with a 3-week interval between each. Serum samples were collected prior to each immunization by extracting blood from the lateral saphenous vein of the mice. Three days after obtaining serum corresponding to the third immunization, the mice were sedated using isoflurane and exsanguinated via cardiac puncture. To obtain serum, blood was allowed to clot at 37 °C for 1 h, followed by centrifugation at 1500× *g* for 20 min. The supernatant was collected and transferred to a new tube.

### 2.3. Immunization Follow Up

To assess successful immunization of the mice, an ELISA was performed to detect any increases in antibody production from pre-immunization to post-immunization. Plates were coated with each VP8* protein, as well as with BSA and GST as negative controls. For coating, 100 ng of each protein were added to each well in 100 µL of carbonate/bicarbonate buffer and incubated for 1h at 37 °C. The plates were then blocked for 1 h at 37 °C with 200 µL of PBS containing 3% of BSA. After blocking, the plates were washed three times with PBST, then incubated with serum samples that had been diluted 1/100 in PBS containing 0.05% tween 20 (PBS-T) for 1 h at 37 °C. After washing, secondary anti-mouse-HRP antibody was added in a dilution of 1/5000 in PBS-T and incubated 1 h at 37 °C. After incubation, the plates were washed again, followed by color development with Sigma FastOPD reagent following the recommendations of the producer. Antibody levels were measured by absorbance at 492 nm in a Multiskan plate reader (Fischer Scientific, Madrid, Spain). The signal of the controls was subtracted, and experiments were performed in triplicate.

### 2.4. Titration of Serums

The antibody titers of each serum against each of the nine antigens were determined by ELISA. Plates were coated as described above and then incubated with serially diluted serum samples in PBS-T ranging from 1/100 to 1/12,800. For P[8] lineages, the dilutions were also performed from 1/1000 to 1/128,000. Later, secondary anti-mouse-HRP antibody was added in a dilution of 1/5000 in PBS-T. Finally, the microplates were developed with Sigma FastOPD reagent, and the antibody levels were measured by absorbance at 492 nm in a Multiskan plate reader (Fischer Scientific). Serum mixing and ELISA were performed to assess significant differences in serum production between the immunized groups. The antibody titer was considered positive when the absorbance was higher than the BSA signal plus three times the standard deviation. Experiments were performed in triplicate.

### 2.5. Statistical Analysis

A one-way ANOVA test was conducted to evaluate differences in antibody levels against different antigens obtained in the ELISA binding experiments. GraphPad Prism version 6.0 for MacOsx was used for statistical analysis. A *p*-value < 0.05 was considered statistically significant.

## 3. Results

### 3.1. Immunization Follow Up

**Figure 1:**
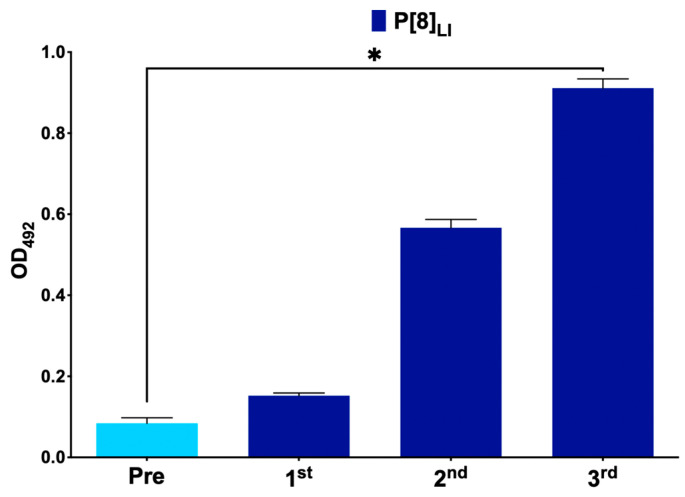
Monitoring of antibody production in group immunized with VP8* P[8]_LI_ after each immunization. Measurements at baseline (day of first immunization), 3 weeks (day 21), 6 weeks (day 42), and 9 weeks (day 63) of antibody production (measured as optical density at 492 nm by enzyme-linked immunosorbent assay—ELISA) in serum (1/100 dilution) against each VP8* P[8]_LI_. Bars show the mean and error bars show the standard deviation. Asterisks represent statistically significant difference between columns 1 and 4.

**Figure 2:**
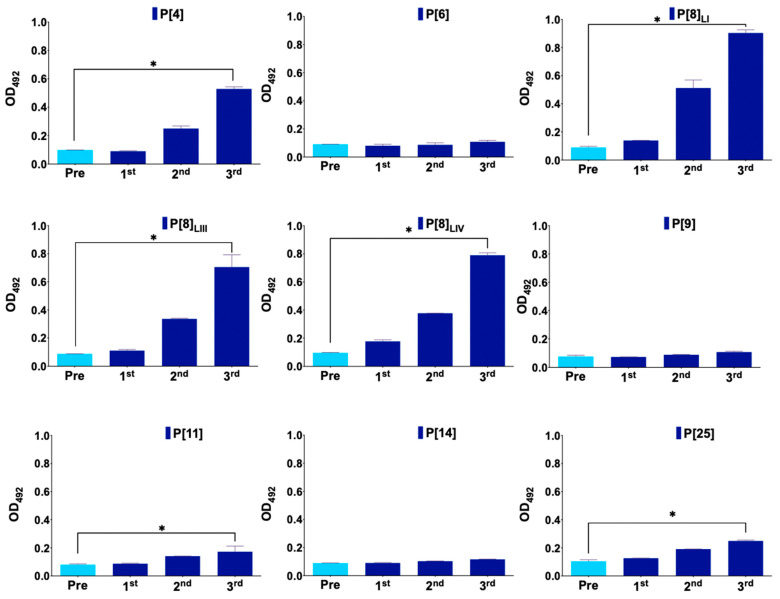
Monitoring of antibody production of mice immunized with nonavalent vaccine after each immunization. Measurements at baseline (day of first immunization), 3 weeks (day 21), 6 weeks (day 42), and 9 weeks (day 63) of antibody production (measured as optical density at 492 nm by enzyme-linked immunosorbent assay—ELISA) in serum (1/100 dilution) against each antigen in the group of mice immunized with the nonavalent vaccine (E1G3). Error bars show standard deviation. Asterisks represent statistically significant difference between columns 1 and 4.

**Figure 3:**
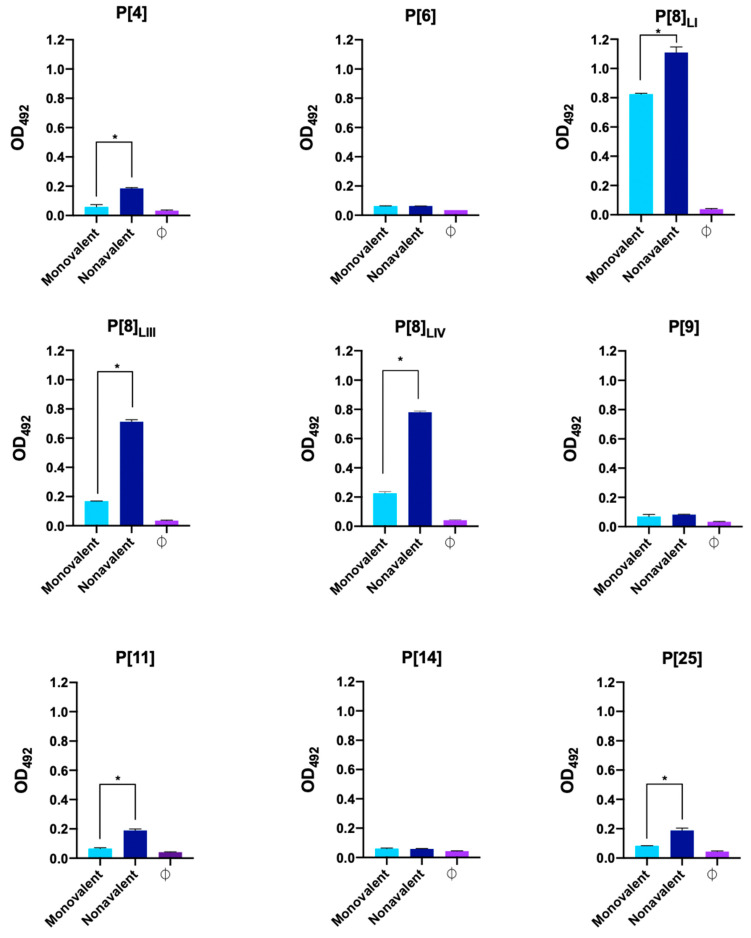
Comparison of antibody production in each group of mice against each antigen. Each graph shows antibody production per group, measured as absorbance at 492 nm by enzyme-linked immunosorbent assay (ELISA) against all antigens at a common dilution (1:100). Error bars show the standard deviation and asterisks indicate significant differences between mice groups immunized with monovalent and nonavalent vaccine.

Figure 1 represents antibody production in mice immunized with the monovalent vaccine, primarily showing antibody production against the vaccine protein VP8*, P[8]_LI_. In the case of the nonavalent vaccine group, antibodies were produced against six proteins, showing statistically significant differences in antibody levels between the first and last serum samples in P[4], P[8]_LI_, P[8]_LII_, P[8]_LIV_, P[11], and P[25] (Figure 2). No antibodies were detected against P[6], P[9], and P[14]. Additionally, a new ELISA was conducted to assess significant differences in antibody production against each antigen between different groups (Figure 3). The results showed no significant differences in antibody production against the VP8*, P[6], P[9], and P[14]. However, the immunization was successful with all the other assayed VP8*s (P[4], P[8]_LI_, P[8]_LIII_, P[8]_LIV_, P[11], and P[25]). In all these cases, the differences in the signal of the group immunized with the nonavalent formulations was significatively higher than that of the group immunized with the monovalent formulation.

### 3.2. Serum Titration

**Figure 4:**
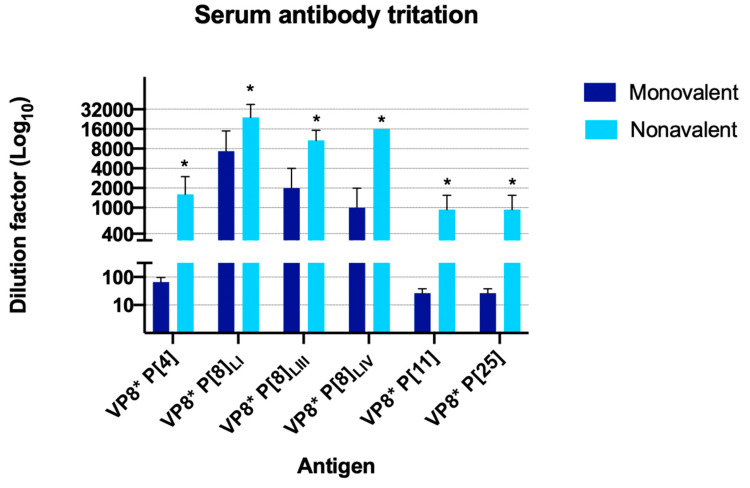
Titration of antibodies against six antigens. The logarithmic scale graph (Log10) shows the mean of the antibody titers by immunization group (monovalent and nonavalent) expressed as the inverse of the dilution at which a higher signal than the control was found against the six antigens against which positive titers had been found. Asterisks indicate significant differences between mice groups.

As observed in Figure 4, mice immunized with the nonavalent vaccine produced high levels of anti-VP8* P[8]_LI_ antibodies (ranging from 1:8000 to 1:32,000). Additionally, they also exhibited elevated antibody titers against other VP8* proteins, P[8] (ranging from 1:8000 to 1:16,000 for lineage III, and 1:16,000 for lineage IV). Furthermore, these mice produced acceptable antibody titers against P[11], P[25] (ranging from 1:400 to 1:1600 in both cases), and P[4] (ranging from 1:800 to 1:3200). In contrast, mice immunized with the monovalent vaccine showed lower antibody titers against the vaccine-specific protein, i.e., anti-VP8* P[8]_LI_ antibodies, ranging from 1:2000 to 1:16,000. Additionally, they exhibited a slight cross-reactivity to antigens P[8]_LIII_ (ranging from 0 to 1:4000) and P[8]_LIV_ (ranging from 0 to 1:2000).

## 4. Discussion

Given the challenges associated with oral RV vaccines, this study developed a parenterally administered candidate based on VP8* protein. Previous studies have emphasized the crucial role of this protein in the early stages of infection by recognizing HBGAs, making the disruption of this recognition critical in preventing infection. Furthermore, these vaccines address many issues associated with oral vaccines and may yield better results in developing countries, where they are most needed. The aim of this study was to demonstrate the immunogenicity of a multivalent vaccine to enhance RV vaccine performance, as RV continues to cause many potentially preventable deaths worldwide despite the introduction of different vaccines. The most relevant result of the present study was being able to obtain high antibody titers to the P[4] genotype and to all the different P[8] lineages assayed with the proposed multivalent formulation. Currently, there are several VP8* vaccines under development, and at least one of them is in a clinical trial where its safety and immunogenicity are being tested in humans [30,32]. This formulation only includes three immunogens, the VP8* from the P[4], P[6] and P[8]_LI_. Interestingly, our results showed that the monovalent vaccines, including a VP8* from the P[8]_LI_, produced antibody titers significantly lower than the other two P[8] lineages tested, P[8]_LIII_ and P[8]_LIV_. This is relevant because various studies have shown a substantial difference between current strains and strains originally cultured or sequenced as the Wa strain. For instance, in the United States, the average difference between all P[8] strains and the original Wa strain has increased as follows: 4% (1974–1980), 5% (1988–1991), and 9% (2005–2013) [33]. This variation is due to point mutations and rearrangement events, which have been favored in recent years due to vaccine introduction. Moreover, significant differences have been reported between the circulating P[8] strains in Belgium, where most belong to lineage III, and the strains in the Rotarix (lineage I) and RotaTeq (lineage II) vaccines [34], similar to the results reported in countries such as India [35], Russia [36], or Japan [15]. This is not exclusive to the first vaccines on the market; if we consider the strains used in the parenteral trivalent non-replicative rotavirus vaccine (NRRV) (Wa-G1P[8]-, DS-1-G2P[4]-, and 1076 G2P[6]), phylogenetic analysis of the globally reported P[8], P[4], and P[6] strains (between 1974 and 2018) shows that 94.9%, 99.8%, and 100%, respectively, belong to lineages not formulated in the vaccine [33]. Overall, these results underscore the need for a broader response against the emergence of mutations and new strains as a direct consequence of vaccine introduction. Furthermore, the nonavalent formulation also succeeded in immunizing the mice against genotypes with a narrower distribution and prevalence, P[11] and P[25] genotypes, showing the potential of a multivalent formulation to include possible emerging genotypes.

Three of the immunogens introduced in the nonavalent formulation, P[6], P[9], and P[14], failed to elicit the production of antibodies. Of these, P[6] is of high importance. Studies conducted in low-income countries such as Mali, Ghana, Kenya, Bangladesh, or Vietnam have reported a high prevalence of genotype P[6], in some cases up to 84.8% [34], highlighting the importance of providing protection against this genotype, especially in developing countries. Furthermore, P[6] has gained additional relevance because nearly all G genotypes observed in nature have been found in combination with the P[4], P[6], and P[8] genotypes, such that a combination of these three P genotypes potentially offer broad protection against all associated G genotypes [37]. Based on this, current vaccine candidates (such as the CDC-6 vaccine) or vaccines in development include a P[6] genotype in their formulation. As for P[9], it is not a very common genotype, although there have been studies showing a temporary emergence in different countries [38]. Regarding P[14], there have been references to its sporadic detection in certain countries such as Honduras [39] and, years ago, in different European countries [40], where it is believed to have originated from recombination with animal strains (such as sheep), explaining its reduced capacity for human infection [31]. However, its prevalence is currently not relevant. 

In addition to the failure to achieve effective immunization against three of the tested genotypes (discussed above), the study also faced the limitation that neutralization assays were not conducted to test neutralization against the nine genotypes or sub-genotypes evaluated here. This was due to the challenge of obtaining nine different replicating rotaviruses, each carrying one of the VP8* proteins. To address this limitation, it would be beneficial to either (a) adapt the various viruses to cell culture, (b) infect cell cultures and/or human intestinal enteroids with non-cell culture adapted viruses, or (c) generate a collection of recombinant viruses through reverse genetics, each carrying the desired P genotype or sub-genotype. Prior to this, the authors will first evaluate new combinations and compositions of VP8*s to achieve high antibody titers against all the desired immunogens.

In summary, this study has shown that, despite the limitations, a multivalent vaccine based in the VP8* protein of rotavirus is feasible and thus will serve as a foundation for future investigations, including new formulations aimed at inducing protection against all the lineages of the most relevant RV genotypes, P[4], P[6], and P[8].

## Data Availability

All data used in this study are included in the manuscript.
